# Effects of Roux-en-Y gastric bypass on fasting and postprandial inflammation-related parameters in obese subjects with normal glucose tolerance and in obese subjects with type 2 diabetes

**DOI:** 10.1186/s13098-015-0012-9

**Published:** 2015-02-24

**Authors:** Kirsten Katrine Lindegaard, Nils Bruun Jorgensen, Rasmus Just, Peter MH Heegaard, Sten Madsbad

**Affiliations:** Zealand Pharma A/S, Smedeland 36, 2600 Glostrup, Denmark; Innate Immunology Group, The National Veterinary Institute, DTU, 1870 Frederiksberg, Denmark; Department of Endocrinology, Hvidovre Hospital, Kettegård Alle 30, 2650 Hvidovre, Denmark

**Keywords:** RYGB, Weight loss, Type 2 diabetes, Glucose tolerant subjects, Inflammatory cytokines

## Abstract

**Background:**

Obesity is characterized by low grade inflammation and an altered secretion of inflammatory cytokines from the adipose tissue. Weight loss has shown to reduce inflammation; however, changes in cytokine profiles during massive weight loss are not well described. The present study explored the hypothesis that Roux-en-Y gastric bypass (RYGB) reduces circulating levels of pro-inflammatory cytokines, while increasing anti-inflammatory cytokines in obese subjects with type 2 diabetes (T2D) and in obese normal glucose tolerant (NGT) subjects.

**Methods:**

Thirteen obese subjects with T2D [weight; 129 ± 14 kg, glycated hemoglobin (HbA1c); 7.0 ± 0.9%, body mass index (BMI); 43.2 ± 5.3 kg/m^2^, mean ± SD] and twelve matched obese NGT subjects [weight; 127 ± 15 kg, HbA1c; 5.5 ± 0.4%, BMI; 41.5 ± 4.8 kg/m^2^, mean ± SD] were examined before, one week, three months, and one year after surgery. Interleukin (IL)-6, leptin, adiponectin, IL-8, transforming growth factor beta (TGF-β), and the incretin hormone glucagon-like peptide-1 (GLP-1) were measured in the fasting state and during a liquid meal. Insulin resistance was evaluated by HOMA-IR.

**Results:**

Weight loss did not differ between the two groups. Before surgery, HbA1c was higher and HOMA-IR lower in T2D patients, however, converged to the values of NGT subjects one year after surgery. Circulating cytokine concentrations did not differ between the two groups at any time point. One week after surgery, circulating IL-6 and IL-8 were increased, while adiponectin and leptin were reduced compared with pre-surgical concentrations. Three months after surgery, IL-8 was increased, leptin was reduced, and no change was observed for IL-6, TGF-β, and adiponectin. One year after surgery, concentrations of IL-6, TGF-β, and leptin were significantly reduced compared to before surgery, while adiponectin was significantly increased.

**Conclusions:**

One year after RYGB, fasting concentrations of IL-6 and leptin were reduced, while no changes were observed in IL-8. TGF-β was decreased and adiponectin increased in both T2D and NGT obese subjects. This study is the first to examine IL-8 and TGF-β in obese subject after RYGB. Resolution of inflammation could offer a potential explanation for the health improvement associated with major weight loss after bariatric surgery.

**Trial registration:**

http://www.clinicaltrials.gov (NCT01579981).

## Background

Obesity is associated with chronic low-grade inflammation, which is thought to be involved in the development of obesity-related co-morbidities, such as insulin resistance, type 2 diabetes (T2D) and cardiovascular diseases (CVD) [1].

The adipose tissue is considered the prime organ for mediating obesity-induced inflammation by secreting high levels of pro-inflammatory molecules including interleukin (IL)-1, IL-6, and tumor necrosis factor-α (TNF-α) secreted by immune cells residing in the stromal vascular fraction. This is thought to contribute to the development of insulin resistance and deterioration of glucose homeostasis [2-4]. Leptin is primarily produced by adipocytes and plasma levels are elevated in obese subjects [5]. Leptin is a central mediator in regulating food intake and energy expenditure [6] and has been shown to inhibit insulin secretion from pancreatic beta cells [7]. In addition, studies have indicated an immunological role for leptin by inducing production of IL-6 and other acute-phase reactants [8,9]. Adiponectin is also produced predominantly by adipocytes, and plasma levels of adiponectin are inversely correlated with the body mass index (BMI) [10,11]. Decreased levels of adiponectin have been linked to impaired insulin sensitivity and increased risk of CVD [12,13]. The chemokine IL-8 has been found to be produced and released from human adipocytes [14], in addition to playing a central role in inflammation, including atherosclerotic processes as a neutrophil chemoattractant [15,16]. Circulating levels of IL-8 are increased in obesity [17,18], in type 1 diabetes (T1D) and T2D patients compared to lean healthy subjects [19] and compared to obese normal glucose tolerance (NGT) subjects [20,21]. Transforming growth factor beta (TGF-β), an immunoregulatory cytokine, has also been found to be associated with obesity and T2D [22-25].

Alterations in cytokine concentrations in NGT subjects and in subjects with impaired fasting glucose, impaired glucose tolerance, and T2D, respectively, have been investigated by Tönjes and co-workers [26]. In support of adipose tissue dysfunction as an early pathogenic event in the development of T2D, the authors reported that pre-diabetic states including impaired fasting glucose and impaired glucose tolerance are associated with increased concentrations of a range of inflammatory cytokines secreted from the adipose tissue, such as chemerin, fetuin-A and retinol binding protein 4 (RBP4) and a decrease in adiponectin, compared to NGT subjects. Comparing an obese group of NGT subjects to an obese T2D group, the same authors found increases in chemerin, fetuin-A, RBP4, leptin and IL-6, as well as a decrease in adiponectin in the latter group, supporting an association between insulin resistance and increased concentration of circulating inflammation-related parameters [26].

Several studies in obese subjects have reported dietary-induced weight loss to improve insulin sensitivity, increase adiponectin levels and reduce circulating levels of leptin, IL-8, and IL-6 [27-30]. Bariatric surgery, including Roux-en-Y gastric bypass (RYGB), restores hepatic and peripheral insulin sensitivity and often causes remission of T2D in most cases before any substantial weight loss is achieved [31,32]. There is a marked increase in hepatic insulin sensitivity within one week post-surgery, while peripheral insulin sensitivity is only improved months post-surgery after weight loss [33]. Several studies have shown that bariatric surgery decreases fasting plasma leptin [34-39] and IL-6 [40-43], while increasing levels of adiponectin independently of weight reduction [36,44].

Whether a more favorable inflammatory profile is linked to the immediate improvement of insulin resistance and resolution of T2D after RYGB is not known. In the present study, we explore the hypothesis that the RYGB-induced improvement in insulin resistance is associated with decreasing pro-inflammatory and increasing anti-inflammatory circulating cytokine levels. We examined short- (one week and three months) and 1-year changes of IL-6, leptin, adiponectin, IL-8, and TGF-β before and after RYGB surgery in obese patients with T2D and in obese NGT subjects. We hypothesized that the fasting levels of pro-inflammatory cytokines would be higher in the T2D group before surgery compared to the NGT group, and that the levels in both groups would decrease and equalize after surgery leading to a more anti-inflammatory profile in both groups.

Recent work in rodents has shown that glucagon-like peptide-1 (GLP-1) can be induced by a variety of inflammatory stimuli, including endotoxin, IL-1, and IL-6, and in a human study, circulating GLP-1 concentrations were increased under inflammatory conditions and correlated with inflammatory markers including IL-6 [45,46]. Since postprandial GLP-1 secretion is increased in RYGB-operated patients [38,47], we studied levels of inflammatory markers in relation to GLP-1 responses both in the fasting and in the postprandial period.

## Methods

### Study population

Study population and study design have been described before [47]. Briefly, thirteen obese subjects with T2D and twelve matched obese NGT subjects were recruited from the Hvidovre Hospital bariatric surgery program (Hvidovre, Denmark). They all met the Danish criteria for bariatric surgery (age > 20 years and BMI > 40 kg/m2 or > 35 kg/m2 with comorbid conditions such as T2D or hypertension), and had accomplished a mandatory preoperative 8% diet-induced total body weight loss before inclusion. Patients were excluded if they had been receiving incretin-based therapies or insulin, anti-thyroid medication or anorectic agents within three months prior to the study.

### Study design and experimental protocol

Participants were examined before and at one week, three months, and one year postoperatively. Antidiabetic medication was paused in diabetic subjects 72 h prior to the preoperative meal test. After surgery, none of the T2D subjects received any antidiabetic medication. On each study day after a 12-h fast, participants were weighed (Tanita, Tokyo, Japan) and then subjected to a liquid meal test consisting of 200 ml of Fresubin Energy Drink [300 kcal, carbohydrate (E% 50), protein (E% 15), fat (E% 35), Fresenius Kabi Deutschland, Bad Homburg, Germany]. The meal was ingested over a 30-min period before and at all study visits after RYGB to avoid dumping. Blood was sampled from a catheter in an antecubital vein at −10, −5, 0, +15, +30, +45, +60, +90, +120, +180, and +240 min compared to the start of the meal. In the present study, only samples collected at time 0, 45, 60, 120 and 240 min were analyzed, and sample at time 0 min was used as fasting baseline level.

### Surgical procedure

Surgery was performed at the Department of Gastroenterology at Hvidovre Hospital (Hvidovre, Denmark) as previously described [39].

### Sample collection and laboratory analysis

Blood samples were collected in clot activator tubes or EDTA tubes. Determination of plasma insulin, GLP-1 and glucose was carried out as described in [47]. HbA1c was determined using HPLC (Tosoh Bioscience, Tokyo, Japan). For protein analyses, venous blood was sampled into EDTA tubes and kept on ice until centrifuged at 4°C, 10 min, 2000 *g*. Plasma aliquots were stored at −80°C until analyzed. Total plasma concentrations of inflammatory markers were determined by electrochemiluminescent sandwich immunoassays (Meso Scale Discovery (MSD), Gaithersburg, MD, USA) enabling simultaneous quantification of multiple analytes. The assays were performed according to the manufacturer’s protocol using an MSD instrument (SECTOR Imager 2400, MSD). All samples for each individual subject were run on the same assay plate and the same batch of kits was applied for all samples. Samples with values greater than the upper limit of quantification were diluted and reanalyzed. In the fasting state IL-6, leptin, adiponectin, IL-8, and TGF-β were measured, and in the postprandial samples IL-6, leptin, adiponectin were measured.

### Statistical analyses

Insulin resistance was calculated using the homeostasis model assessment of insulin resistance (HOMA-IR) as Insulin_fasting_ × Glucose_fasting_/(22.5 × 6.945). To assess the possible effect of RYGB on the postprandial cytokine response, total AUC for IL-6, adiponectin, and leptin were calculated by using the trapezoidal method (GraphPad Prism v. 4). Statistical analyses were carried out using Mann–Whitney *U*-test for comparisons between groups. For comparisons of pre- and post-surgical time-points, Friedman’s nonparametric repeated measures ANOVA with Dunn’s multiple comparison post-hoc test was applied and included only patients with complete data set (blood sample drawn at all four study time-points). Relationship between variables was assessed by non-parametric Spearman rank correlation. Statistical analysis was performed using GraphPad Prism v. 4 (San Diego, CA, USA). The level of significance was set at *P* < 0.05.

### Ethics statement

Written informed consent was obtained from all participants. The study was approved by the Municipal Ethical Committee of Copenhagen (Reg. nr. H-A-2008-080-31742) and was in accordance with the Helsinki-II declaration and the Danish Data Protection Agency. The study was registered at http://www.clinicaltrials.gov (NCT01579981).

## Results

### Study population

**Figure 1:**
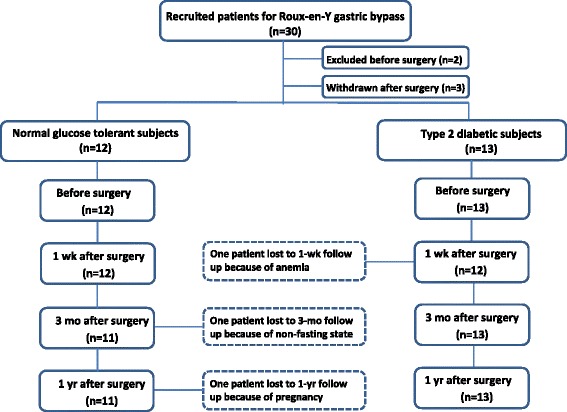
**Trial profile.** Numbers of subjects initially enrolled are depicted and subject disposition (number of T2D vs NGT subjects, subjects who were excluded or withdrawn, and subjects who were lost to follow up) is described.

Overview of enrolled patients is depicted in Figure 1. A total number of 30 patients were included in the study of which two were not operated and three subjects did not wish to participate following surgery. One subject with T2D could not be studied one week after RYGB because of anemia. One NGT subject was excluded from the three-month follow-up data set, due to excessively high fasting insulin and C-peptide concentrations, indicating a non-fasting state. One NGT subject was not examined at the one-year follow-up due to pregnancy (Figure 1). Before surgery, twelve subjects with T2D were treated with ≥ 1 oral antidiabetic medication, and one subject was diet-treated only. After RYGB, none of the T2D patients received any anti-diabetic medication.

### Changes in weight and glucose metabolism

Data are presented in Table 1. The most important findings were that RYGB-induced weight loss in T2D subjects: one week: 2.1% ± 1.6%, three months: 13.2% ± 3.7%, one year: 22.2% ± 8.8% and in NGT subjects: one week: 2.5% ± 1.3%, three months: 15% ± 4.5%, and one year: 25.2% ± 8.0%. Body weight and BMI did not differ between the two groups before or after surgery. Changes in fasting glucose and insulin concentrations after surgery are given in Table 1. Before surgery, HbA1c levels were lower in NGT subjects compared to T2D subjects (*P* = 0.0002), but three months and one year after surgery, HbA1c levels did not differ significantly between the groups (three months: *P* = 0.06, one year: *P* = 0.24) and HbA1c values were significantly reduced in T2D subjects at the one-year follow-up (*P* = 0.0007), while no changes occurred in NGT subjects. HOMA-IR was lower in the NGT group before surgery (*P* = 0.002), and one week (*P* = 0.001), and three months (*P* = 0.05) after surgery compared to the T2D group, but the levels did not differ between the two groups at the one-year follow-up.

**Table 1:** **Characteristics of type 2 diabetic subjects and normal glucose tolerant subjects**

		**Pre-RYGB**	**1 week**	**3 months**	**1 year**	* ***P*** **value**
**Weight (kg)**	*N* (T2D; NGT)	13; 12	12; 12	13; 11	13; 11	
	T2D	129 ± 14	127 ± 13	112 ± 15***	101 ± 20***	*<0.0001*
	NGT	127 ± 15	124 ± 15	106 ± 13**	95 ± 16***	*<0.0001*
	†*P* value	0.57	0.29	0.25	0.49	
**BMI (kg/m** ^2^ **)**	*N* (T2D; NGT)	13; 12	12; 12	13; 11	13; 11	
	T2D	43.2 ± 5.3	42.5 ± 5.5	37.2 ± 5.8***	34.8 ± 7.9***	*<0.0001*
	NGT	41.5 ± 4.8	40.5 ± 4.5	34.4 ± 3.4**	31.3 ± 4.8***	*<0.0001*
	†*P* value	0.34	0.4	0.12	0.42	
**Glucose (mmol/L)**	*N* (T2D; NGT)	13; 12	12; 12	13; 11	13; 11	
	T2D	8.9 ± 2.3	7.0 ± 1.1*	6.9 ± 1.6**	6.3 ± 1.6***	*0.0001*
	NGT	5.5 ± 0.7^†††^	5.0 ± 0.6^†††^	4.9 ± 0.4**^†††^	5.0 ± 0.3^††^	*0.01*
	†*P* value	*<0.0001*	*0.0002*	*0.001*	*0.007*	
**HbA1c (%)**	*N* (T2D; NGT)	13; 12		13; 11	13; 10	
	T2D	7.0 ± 0.9		5.9 ± 0.8	5.6 ± 0.6***	*0.0007*
	NGT	5.5 ± 0.4^†††^		5.4 ± 0.3	5.4 ± 0.3	0.22
	†*P* value	*0.0002*		0.06	0.24	
**Insulin (mmol/L)**	*N* (T2D; NGT)	13; 12	12; 12	13; 11	13; 11	
	T2D	125.0 ± 77.0	72.9 ± 32.1*	58.1 ± 35.4***	46.7 ± 27.1***	*<0.0001*
	NGT	82.2 ± 28.3	49.4 ± 13.5	42.8 ± 13.8**	35.8 ± 16.5***	*0.0001*
	†*P* value	0.29	0.053	0.6	0.45	
**HOMA-IR**	*N* (T2D; NGT)	13; 12	12; 12	13; 11	13; 11	
	T2D	6.6 ± 3.6	3.2 ± 1.5*	2.5 ± 1.7**	1.8 ± 1.1***	*<0.0001*
	NGT	2.9 ± 1.1^††^	1.6 ± 0.5^††^	1.3 ± 0.5*^†^	1.1 ± 0.5***	*0.0002*
	†*P* value	*0.002*	*0.001*	*0.047*	0.11	

Characteristics of T2D subjects and NGT subjects before (Pre-RYGB), 1 week, 3 months, and 1 year after Roux-en-Y gastric bypass. Data are presented as mean ± SD. BMI, body mass index; HbA1c, glycated hemoglobin; HOMA-IR, homeostasis model assessment of insulin resistance; NGT, normal glucose tolerance; T2D, type 2 diabetes. ^*^ *P*-value for overall comparison of time-points within groups, *P < 0.05, **P < 0.01, ***P < 0.0001; †*P*-value for comparison between groups, ^†^ *P* < 0.05, ^††^ *P* < 0.01, ^†††^ *P* < 0.0001.

### Fasting concentrations of inflammatory cytokines and GLP-1

**Figure 2:**
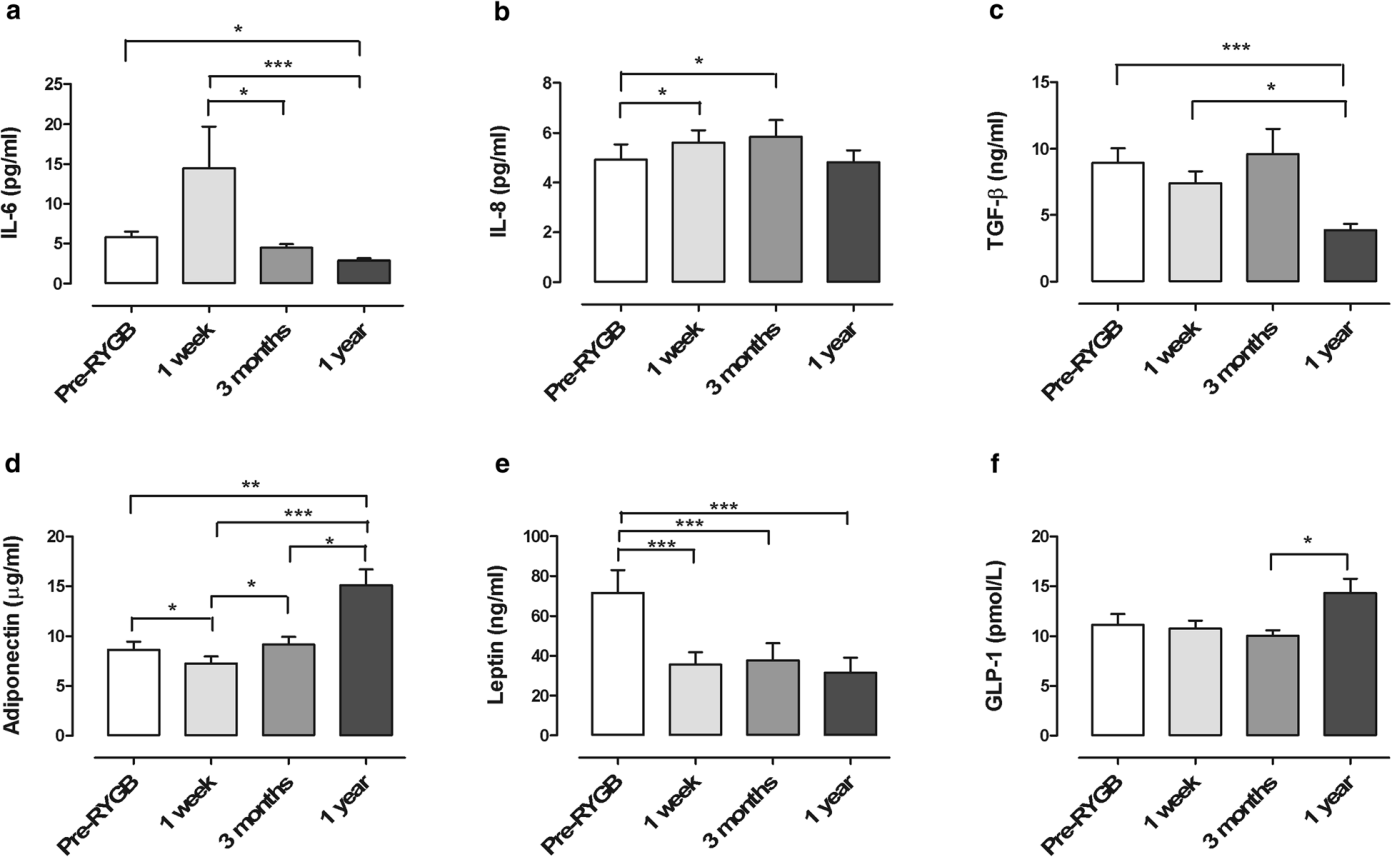
**a-f Fasting plasma concentrations of inflammatory cytokines and GLP.** Fasting plasma concentrations of **(a)** IL-6 (*N* = 18), **(b)** IL-8 (*N* = 18), **(c)** TGF-β (*N* = 22), **(d)** adiponectin (*N* = 20), **(e)** leptin (*N* = 18), and **(f)** GLP-1 (*N* = 21) before (Pre-RYGB), one week, three months, and one year after Roux-en-Y gastric bypass in the whole study population. Data are presented as mean ± SEM. Only patients with complete fasting data set (samples drawn at all four time points) were included. Comparisons between time-points was made using ANOVA (non-parametric Friedman test for repeated measures) followed by Dunn’s multiple test for statistical differences, *P < 0.05, **P < 0.01, ***P < 0.001.

Data are presented in Figure 2. Considering the whole study population, fasting circulating IL-6 and IL-8 levels were increased one week after surgery, but only IL-8 reached statistical significance. At the same time point, adiponectin and leptin levels were reduced compared with pre-surgical concentrations. TGF-β levels did not differ before and one week after surgery. At the three-month follow-up, IL-8 was increased, leptin was reduced, and no changes occurred in IL-6, TGF-β, and adiponectin compared to before surgery. One year after surgery, however, concentrations of IL-6, TGF-β and leptin were significantly reduced compared to before surgery, while adiponectin was increased. IL-8 did not differ from before and one year after surgery. We did not find any significant differences in cytokine concentrations between the two groups at any of the four study time-points (data not shown).

Table 2 illustrates relevant correlations between cytokine and clinical measures performed on 1-year changes for the whole study population and in the two groups separately. The decrease in IL-6 and TGF-β correlated with improved HbA1c in the T2D group. The increase in adiponectin concentrations correlated with the decrease in leptin. Leptin changes were positively associated with BMI and HOMA-IR only in the NGT group. Changes in adiponectin and IL-8 correlated positively with GLP-1 in the NGT group (Table 2).

**Table 2:** **Correlation coefficients of 1-year changes**

**Variable 1**	**Variable 2**	**Whole group** ***r*** **(** ***N; P*** **)**	**NGT group** ***r*** **(** ***N; P*** **)**	**T2D group** ***r*** **(** ***N; P*** **)**
**IL-6**	BMI	0.16(20; 0.49)	0.01(9; 0.98)	0.28(11; 0.40)
	HOMA-IR	0.19(20; 0.19)	−0.07(9; 0.88)	0.21(11; 0.52)
	HbA1c	*0.47(19; 0.04)*	0.02(8; 0.98)	*0.82(11; 0.002)*
	TGF-β	*0.65(20; 0.002)*	*0.70(9; 0.04)*	*0.71(11; 0.01)*
**IL-8**	BMI	0.02(20; 0.92)	−0.13(9; 0.73)	0.03(11; 0.94)
	HOMA-IR	0.06(20; 0.81)	−0.20(9; 0.61)	0.37(11; 0.26)
	HbA1c	−0.14(19; 0.57)	0.15(8; 0.73)	0.45(11; 0.17)
	GLP-1	*0.59(20; 0.007)*	*0.80(9; 0.01)*	0.54(11; 0.09)
	Adiponectin	0.35(20; 0.12)	*0.67(9; 0.05)*	0.27(11; 0.42)
**TGF-β**	BMI	0.28(24; 0.19)	0.37(11; 0.26)	0.25(13; 0.42)
	HOMA-IR	0.05(24; 0.81)	−0.10(11; 0.77)	0.19(13; 0.54)
	HbA1c	0.25(23; 0.26)	0.07(10; 0.84)	*0.64(13; 0.02)*
**Adiponectin**	BMI	−0.40(21; 0.07)	−0.28(9; 0.11)	−0.42(12; 0.17)
	HOMA-IR	−0.28(21; 0.21)	−0.57(9; 0.11)	−0.19(12; 0.56)
	HbA1c	−0.06(20; 0.79)	−0.07(8; 0.86)	−0.32(12; 0.31)
	GLP-1	*0.47(21; 0.03)*	*0.87(9; 0.003)*	0.16(12; 0.62)
	Leptin	*−0.58(21; 0.01)*	*−0.68(9; 0.04)*	−0.36(12; 0.26)
**Leptin**	BMI	*0.65(21; 0.002)*	*0.75(9; 0.02)*	0.56(12; 0.06)
	HOMA-IR	0.36(21; 0.11)	*0.72(9; 0.03)*	0.45(12; 0.14)
	HbA1c	−0.14(20; 0.55)	0.41(8; 0.31)	−0.08(12; 0.19)
	Insulin	*0.51(21; 0.02)*	0.28(9; 0.46)	0.41(12; 0.81)

Clinically relevant significant and non-significant correlation coefficients in the whole study population and in T2D subjects and NGT subjects. Data are presented as *r*: correlation coefficient of correlation, and the sample size and *P*-value in parentheses. NGT, normal glucose tolerant subjects; T2D, type 2 diabetes subjects; BMI, body mass index; HbA1c, glycated hemoglobin; HOMA-IR, homeostasis model assessment of insulin resistance. Data are analyzed by Spearman correlation test, *P* < 0.05.

### Postprandial concentrations of inflammatory cytokines and GLP-1

**Figure 3:**
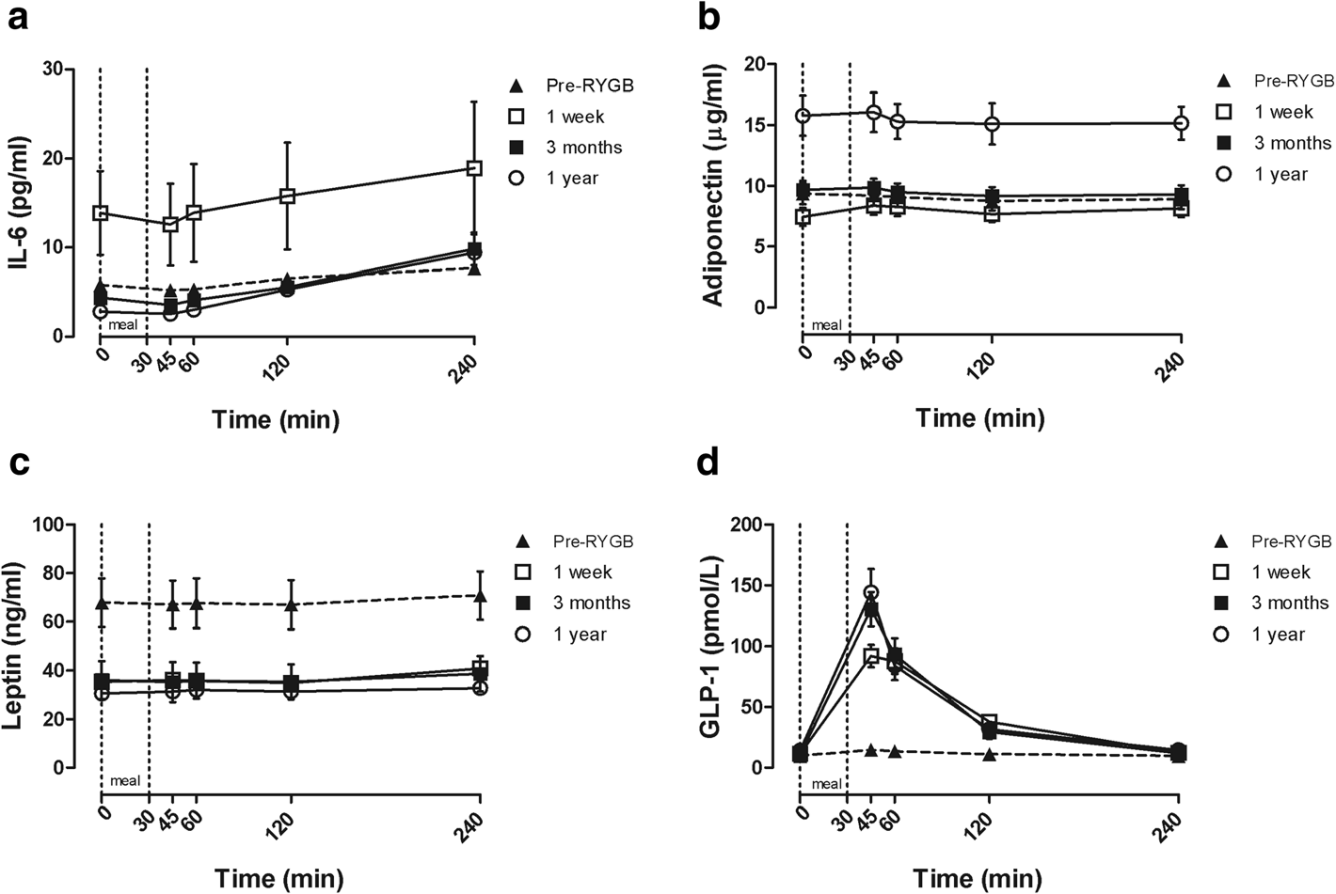
**Postprandial cytokine and GLP-1 response in the whole study population.** Postprandial response of **(a)** IL-6, **(b)** adiponectin, **(c)** leptin, and **(d)** GLP-1 in the whole study population before (Pre-RYGB) (*N* = 21), one week (*N* = 20), three months (*N* = 20), and one year (*N* = 20) after Roux-en-Y gastric bypass in the whole study population. Data are presented as mean ± SEM.

The postprandial responses of cytokines and GLP-1 are presented in Figure 3a-d and depicted as total AUC in Table 3. Total AUC for the different cytokines in T2D patients and NGT subjects did not differ significantly at any study time-point and the two groups will be analyzed as one group. An increase in total AUC for IL-6 was observed one week after surgery followed by a decrease to preoperative response levels after three months and after one year (Figure 3a). Total AUC for adiponectin was significantly increased one year after surgery compared with both before, one week, and three months after surgery (*P* < 0.0001) (Figure 3b). At all post-operative follow-ups, a significant reduction in leptin was observed with no significant differences between the responses after surgery (Figure 3c) (*P* < 0.0001). Postprandial GLP-1 responses were markedly increased at all three post-surgical follow-ups (Figure 3d) (*P* < 0.0001). Correlation analysis revealed no associations between the postprandial GLP-1 response and postprandial inflammatory cytokine responses before or after RYGB surgery (data not shown).

**Table 3:** **Total AUC for IL-6, adiponectin, leptin and GLP-1 in response to meal intake**

**Total AUC**	**Mean (95% CI)**	***P*** **-value**
**IL-6 (pg/ml × 240 min)** ***N*** **= 18**		*0.001*
Pre-RYGB	1525 (1151; 1899)	
1 week	3897 (530; 7264)	
3 months	1471 (1099; 1843)	
1 year	1312 (803; 1821)^b^	
**Adiponectin (μg/ml × 240 min)** ***N*** **= 18**		*<0.0001*
Pre-RYGB	2006 (1615; 2398)	
1 week	1891 (1505; 2278)	
3 months	2188 (1801; 2575)^b^	
1 year	3541 (2821; 4261)^a,b,c^	
**Leptin (ng/ml × 240 min)** ***N*** **= 18**		*<0.0001*
Pre-RYGB	16997 (11138; 22856)	
1 week	8883 (5600; 12166)^a^	
3 months	9113 (4981; 13245)^a^	
1 year	7883 (3904; 11862)^a^	
**GLP-1 (pmol/l × 240 min)** ***N*** **= 21**		*<0.0001*
Pre-RYGB	2742 (2433; 3051)	
1 week	10257 (7962; 12551)^a^	
3 months	11606 (8833; 14379)^a^	
1 year	11642 (8398; 14886)^a^	

Total AUC for IL-6 (*N* = 18), adiponectin (*N* = 18), leptin (*N* = 18), and GLP-1 (*N* = 21) in response to meal intake measured in the whole study population before (Pre-RYGB), 1 week, 3 months, and 1 year after Roux-en-Y gastric bypass. Data are presented as mean with 95% confidence interval (CI). Comparisons between time-points was made using ANOVA (non-parametric Friedman test for repeated measures) followed by Dunn’s multiple test for statistical differences, ^a^different from pre-surgical levels, ^b^different form one-week level, and ^c^different from one year level, *P* < 0.05.

## Discussion

In the present study, we have assessed fasting and postprandial responses of IL-6, IL-8, TGF-β, leptin, and adiponectin before as well as one week, three months, and one year after RYGB in 13 obese patients with T2D and in 12 matched obese NGT subjects. Our main findings were that RYGB induces changes in circulating concentrations of cytokines in both obese T2D patients and in obese NGT subjects by decreasing fasting plasma concentrations of the pro-inflammatory cytokine IL-6, leptin, and the regulatory cytokine TGF-β, and by increasing the anti-inflammatory adiponectin. We found an increase in IL-8 concentrations at one week and at three months after RYGB, but one year after surgery the concentrations had returned to preoperative levels.

Previous RYGB studies have focused primarily on well-characterized pro-inflammatory markers and have rarely prospectively examined acute and up to 1-year changes after surgery of both pro- and anti-inflammatory markers. The present study is the first to examine the pro-inflammatory cytokine IL-8 and the regulatory cytokine TGF-β after RYGB. To our knowledge, only one other study has investigated the postprandial response of cytokines following bariatric surgery [48].

### Fasting cytokine response

We hypothesized that T2D subjects would have a higher inflammatory burden as reflected in a more inflammatory circulatory cytokine profile. We observed that pre-surgical IL-6 concentrations in the T2D group were higher compared to NGT subjects, although not significantly so, which also has been shown in other studies [40,49]. Significantly higher IL-6 concentrations have been found in T2D subjects when compared to healthy obese subjects [50], which, in relation to the decrease in IL-6 we observe one year after RYGB associated with improvement of glucose metabolism, may imply a role of IL-6 in glycemic control when insulin resistance is present. However, a clear conclusion about causality cannot be obtained from the present study and the role of IL-6 in T2D and in insulin resistance remains debatable [51].

Despite earlier findings that impaired insulin sensitivity is associated with changes in a range of inflammatory markers including RBP4, IL-6, leptin, and adiponectin [26] we were unable to show differences in fasting concentrations of cytokines between T2D patients and NGT subjects at any study time-point. This may in part be explained by the mandatory preoperative 8% diet-induced total weight loss before surgery and that both groups displayed morbid obesity with a mean BMI of about 42 kg/m^2^. Nevertheless, insulin resistance evaluated by HOMA-IR was more pronounced in T2D patients before and the first three months after surgery. One year after RYGB, insulin resistance did not differ between the two experimental groups. The present study was, however, not designed to investigate the relative role of the inflammatory parameters in relation to insulin resistance, insulin secretion, and development of T2D.

The reductions in IL-6 concentrations we report one year after RYGB is in agreement with some other RYGB studies [41,42] and consistent with other weight loss intervention studies reporting 1-year follow-ups, either through dietary restriction [30] or other types of bariatric surgery procedures [40,43,49]. Other studies have reported decrease in IL-6 within six months after surgery [52], while other investigators have not been able to detect any changes [34,36,53,54].

The increase in fasting concentrations of the anti-inflammatory adiponectin is consistent with the findings of a number of RYGB studies [34,36,44]. Brethauer and co-workers found an increase in adiponectin appearing three months after surgery [34], while Trakhtenbroit and co-workers report lack of increase until two years after RYGB with no changes observed in a cohort of T2D subjects undergoing gastric banding. Subjects in the RYGB group lost significantly more fat mass than the gastric banding group [55].

The decrease in adiponectin we observe immediately after surgery, has, to our knowledge, not been reported before. In a study with obese subjects undergoing liposuction, decreases in adiponectin in addition to increases in IL-6 was reported and was suggested to indicate that IL-6 suppresses adiponectin secretion [56]. *In vivo* studies in obese subjects have shown that low levels of plasma adiponectin were inversely correlated with circulating IL-6 [28,57]. While *in vitro* studies have demonstrated downregulation of adiponectin mRNA in adipose tissue fragments incubated with IL-6 [28,58], adiponectin has also been shown to increase insulin sensitivity through an IL-6-dependent pathway [59]. We did, however, not observe any correlation between changes in adiponectin concentrations and in IL-6, IL-8, or TGF-β levels or in HOMA-IR. Thus, our results do not support a role for adiponectin as mediator of the improved insulin sensitivity early after surgery.

The changes in adiponectin were negatively associated with leptin in the NGT group only. There is a well-reported inverse relationship between adiponectin and leptin as they are both secreted and regulated by the adipose tissue, so we would have expected this correlation to be present in both groups.

We found a reduction in circulating leptin as early as one week after surgery and before significant changes in body weight; a finding which is supported by Isbell and co-workers and others with two- and three-week post-surgical follow-ups [35,37,39,60,61]. Reduction of circulating leptin during caloric restriction has been suggested to be an essential component of the neuroendocrine response to fasting [62,63], and the immediate decrease in leptin could therefore potentially be ascribed to post-surgical caloric restriction rather than the RYGB procedure itself. The decrease in leptin concentrations one year after surgery was associated with improved BMI measures and HOMA-IR in the NGT group only.

Lack of effect on inflammation-related circulating cytokines after RYGB could be explained by the short duration of some studies, and may suggest that either a certain amount of weight loss, a period of weight stabilization or improvement of metabolic instability is required before changes in plasma concentrations are manifested. The maximal weight loss after RYGB is obtained between 1–1.5 year after surgery [64], and evidence seems to suggest that at least two years is required post-surgery for stabilization of the inflammatory profile [65]. In addition, the surgical procedure and subsequent healing process might elicit an inflammatory response delaying the weight-induced reduction in inflammatory cytokines until several months after surgery. Another explanation for a delay in reduction of pro-inflammatory mediators may be that the restrictive diet following surgery resembles a state of starvation, which has been shown to initiate certain pro-inflammatory responses [66].

To our knowledge, the response of the pro-inflammatory cytokine IL-8 and the regulatory TGF-β has not previously been studied in relation to bariatric surgery. The observed increase in IL-8 after RYGB is of interest as this chemokine potentially is involved in the pathogenesis of atherosclerosis and CVD [16], and thus, we would have expected that a health promoting intervention like RYGB should result in a lowering of IL-8 levels. Similar to our finding, Bruun and coworkers reported an increase in IL-8 after 20 weeks and 24 weeks, respectively, of dietary-induced weight loss of approximately 15% [17,27]. Other cell types besides adipocytes involved in acute and chronic inflammatory responses could add to the secretion of IL-8 [67] and this could evidently also be the case after acute weight loss. In addition, other substances released from the adipose tissue during weight loss, such as organochlorine compounds, may also stimulate the release of IL-8 [68] and of other adipose tissue-derived cytokines [69]. The immediate increase after RYGB could also be a surgery-related stress response, as recently suggested [70].

Studies measuring circulating TGF-β are somewhat limited due to TGF-β being present in an inactive form requiring proteolytic activation. Furthermore, TGF-β is released during the coagulation process complicating comparisons between measurements obtained in serum versus plasma [71]. These analytic difficulties might explain to some degree the diverging conclusions on TGF-β in diabetes research. Our data on TGF-β suggest elevated levels in the obese state as the RYGB-induced weight loss induced a decrease in circulatory TGF-β concentrations. Herder and co-workers found that enhanced levels of TGF-β were linked to increased risk of T2D [24]. The relevance of a reduction in TGF-β is, however, difficult to evaluate as the influence of weight loss on TGF-β concentrations and the metabolic impact of either increasing or decreasing these concentrations in humans is unknown.

### Postprandial cytokine response

The prandial levels of cytokines followed the changes in fasting plasma levels, and consistent with previous work in obese and/or diabetic subjects no postprandial changes in leptin or adiponectin concentrations were observed [29,72-74]. A small transient decrease in IL-6 concentrations was observed immediately after food intake followed by an increase lasting for the remainder of the meal test reaching final concentrations above baseline. The initial decrease and the following increase in postprandial IL-6 concentrations in obese and/or T2D have been demonstrated before [53,75-79].

IL-6 has been reported to have an effect on the secretion of GLP-1 by intestinal L-cells [45], and circulating levels of GLP-1 has been found to correlate with systemic IL-6 concentrations and other markers of inflammation suggesting an inflammation-dependent regulation of GLP-1 secretion [46]. These finding might indicate a cross-talk between the gut and the immune system. In the present study, we found changes in fasting GLP-1 to be positively associated with IL-8 and with adiponectin, whereas no association between postprandial GLP-1 secretion and IL-6 or other cytokine responses could be demonstrated.

The present study has several limitations; participants were included after a preoperative weight loss of about 8%, likely to improve several metabolic parameters, especially hepatic insulin sensitivity, but could also have affected the inflammatory profile in the two groups. Nevertheless, postoperative metabolic improvements were still observed, although the magnitude probably had been greater if participants had not been subjected to the preoperative weight loss. The study was based on the assumption that a postprandial response would be a more sensitive measure of endocrine changes and therefore the study was powered to take advantage of this, meaning that small postoperative changes in fasting circulatory levels may not reach statistically significance. In the present study, inclusion of more patients could have increased the power of the statistical analysis and strengthened the study. Here we use HOMA-IR which has been shown to correlate well with full body insulin sensitivity [80]. However, in states of caloric restriction, HOMA-IR probably reflects hepatic insulin sensitivity more than whole body insulin sensitivity [81], which makes it difficult to evaluate the relationship between changes in inflammatory markers and changes in hepatic and peripheral muscle and fat tissue insulin sensitivity. Finally, we did not include a control group subjected to the same postoperative diet, which would be of major interest provided that diet-adherence can be controlled. Therefore, as other bariatric surgery studies, the present study lacks a clinically relevant control group representing a comparable non-operative-induced weight loss.

## Conclusion

Low-grade chronic inflammation associated with obesity is considered a major risk factor for the development of obesity-related comorbidities, such as insulin resistance and CVD. Resolution of the unfavorable inflammatory state could offer a potential explanation for the health improvement associated with major weight loss after bariatric surgery. In the present study, short and up to 1-year changes in cytokines and metabolic variables were reported in obese patients with T2D and in obese NGT subjects after RYGB surgery. Pre-operative levels of inflammatory mediators did not differ between the two groups. RYGB reduced fasting and postprandial concentrations of the pro-inflammatory cytokines IL-6, leptin, and the regulatory cytokine TGF-β to a similar extent in both groups. The anti-inflammatory adiponectin was increased, while the pro-inflammatory IL-8 did not differ before and after surgery. Notably, this study is the first to examine and report changes in IL-8 and TGF-β in obese subject after RYGB.

## Abbreviations

CVD: Cardiovascular disease
BMI: Body mass index
RYGB: Roux-en-Y gastric bypass
GLP-1: Glucagon-like peptide-1
HOMA-IR: Model assessment of insulin resistance
HbA1c: Glycated hemoglobin
HPLC: High pressure liquid chromatography
RBP4: Retinol binding protein 4
SEM: Standard area of the mean
SD: Standard deviation
AUC: Area under the curve
IL-: Interleukin-
TGF-β: Transforming growth factor beta-β
TNF-α: Tumor necrosis factor-α
MSD: Meso scale discovery
NGT: Normal glucose tolerant
T2D: Type 2 diabetes
